# The clinical relative biological effectiveness and prostate‐specific antigen kinetics of carbon‐ion radiotherapy in low‐risk prostate cancer

**DOI:** 10.1002/cam4.5045

**Published:** 2022-07-19

**Authors:** Yu‐Mei Kang, Hitoshi Ishikawa, Taku Inaniwa, Yuma Iwai, Naruhiro Matsufuji, Goro Kasuya, Noriyuki Okonogi, Yu‐Ming Liu, Yee Chao, Masaru Wakatsuki, Hirohiko Tsujii, Hiroshi Tsuji

**Affiliations:** ^1^ QST Hospital, National Institutes for Quantum Science and Technology Chiba Japan; ^2^ Division of Radiation Oncology, Department of Oncology Taipei Veterans General Hospital Taipei Taiwan; ^3^ Faculty of Medicine National Yang Ming Chiao Tung University Taipei Taiwan; ^4^ Department of Accelerator and Medical Physics National Institutes for Quantum Science and Technology Chiba Japan; ^5^ Department of Oncology Taipei Veterans General Hospital Taipei Taiwan

**Keywords:** carbon‐ion radiotherapy, low‐risk, prostate cancer, prostate‐specific antigen (PSA), PSA kinetics, relative biological effectiveness (RBE)

## Abstract

**Background:**

To evaluate the clinical relative biological effectiveness (RBE) of carbon‐ion radiotherapy (C‐ion RT) for prostate cancer.

**Methods:**

The records of 262 patients with low‐risk prostate cancer (median age, 65 [47–80] years) treated with C‐ion RT at QST Hospital, National Institutes for Quantum Science and Technology in Japan during 2000–2018 were reviewed retrospectively. Four different protocol outcomes and prostate‐specific antigen (PSA) responses were evaluated. The median follow‐up was 8.4 years. The Kaplan–Meier method was used to estimate the biochemical or clinical failure‐free rate (BCFFR). Clinical RBE was calculated using the tumor control probability model.

**Results:**

The 5‐, 7‐, and 10‐year BCFFRs were 91.7%, 83.8%, and 73.2%, respectively. The 10‐year BCFFRs of patients who received C‐ion RT at 66 Gy (RBE) in 20 fractions, 63 Gy (RBE) in 20 fractions, and 57.6 Gy (RBE) in 16 fractions were 81.4%, 70.9%, and 68.9%, respectively. The PSA level and density during follow‐up were better in the patients treated with the lower fraction size. A higher PSA nadir and shorter time to PSA nadir were risk factors for biochemical or clinical failure by multivariate Cox regression. The tumor control probability analysis showed that the estimated clinical RBE values to achieve an 80% BCFFR at 10 years for 20, 16, and 12 fractions were 2.19 (2.18–2.24), 2.16 (2.14–2.23), and 2.12 (2.09–2.21), respectively.

**Conclusions:**

Using clinical data from low‐risk prostate cancer patients, we showed the clinical RBE of C‐ion RT decreased with increasing dose per fraction.

## Graphical abstract

This is the first study to use clinical data of carbon‐ion radiotherapy from low‐risk prostate cancer to evaluate the clinical relative biological effectiveness (RBE). Our results showed that the RBE of carbon‐ion decreased when the dose per fraction increased.

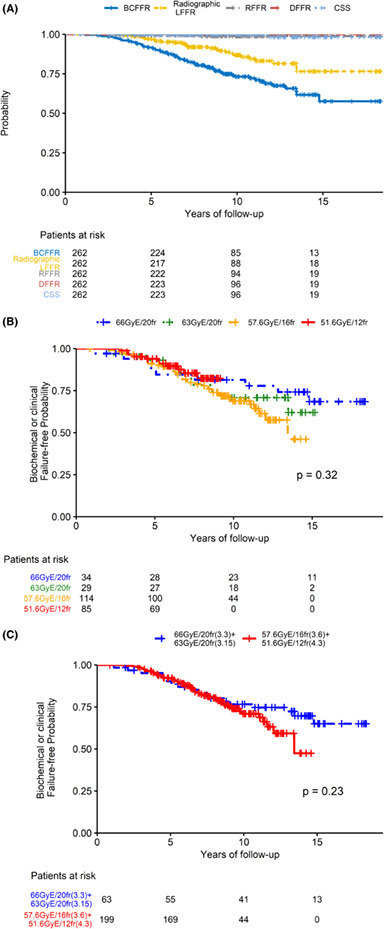

## 1 INTRODUCTION

Prostate cancer is the second most common cancer in males worldwide.^1^ There were approximately 1,276,000 newly diagnosed prostate cancer patients and 359,000 prostate cancer deaths in 2018.^1^ Radiotherapy (RT) is a curative treatment option for prostate cancer.^2^ Carbon‐ion RT (C‐ion RT) is a safe and effective treatment for localized prostate cancer because of its physical and radiobiological advantages,^3^ and it showed excellent PSA control with a low rate of toxicity.^4^, ^5^, ^6^ C‐ion RT is high linear energy transfer radiation, resulting in a high relative biological effectiveness (RBE), which is the ratio of the dose of photons to the dose of C‐ion to achieve the same biological effect.^7^ In Japan, a dose‐independent clinical RBE system has been used in clinical treatments of C‐ion RT, in which a fixed 10% survival of human salivary gland (HSG) tumor cells was selected as a biological endpoint for RBE calculations independent of dose level.^21^ Due to this dose‐independent assumption, the clinical dose of C‐ion beams described by the unit of Gy (RBE) is no more photon equivalent. However, studies evaluating the clinical RBE of the C‐ion beam based on clinical results are lacking.

Low‐risk localized prostate cancer can be managed with active surveillance or curative treatments, such as radical prostatectomy and RT. Androgen deprivation therapy is unnecessary if RT is received.^8^, ^9^ Therefore, low‐risk prostate cancer is ideal for evaluating precise RT outcomes without the interference of systemic medication. For particle therapy, few studies have evaluated the effect that changing the fraction size in clinical treatments has on RBE. However, preclinical studies revealed that RBE actually varies with the fraction size.^10^, ^11^ In this study, we aimed to determine the clinical RBE of C‐ion RT for low‐risk prostate cancer based on clinical results.

The QST Hospital in Chiba, Japan, the former National Institute of Radiological Sciences (NIRS) Hospital, is the pioneer in C‐ion RT, starting the first clinical trial of C‐ion RT for prostate cancer in 1994.^7^, ^12^ Five clinical trials of C‐ion RT for prostate cancer, involving more than 3500 patients, were conducted using different dose and fractionation protocols,^4^, ^12^, ^13^, ^14^, ^15^ and detailed treatment information and long‐term follow‐up results are available. The protocol doses for the clinical trials of 16 and 12 fractions have been determined from the clinically validated protocol doses for 20 fractions under an assumption that the clinical dose of C‐ion beams represented by the unit of Gy (RBE) was photon equivalent with the α/β ratio of 1.5 Gy. This assumption was acceptable for the moderate protocol doses adopted in the clinical trials. However, before starting more advanced hypofractionation schemes with high protocol doses, e.g., 4 fraction treatments, this assumption should be re‐evaluated. This is the first study to use clinical data to evaluate the clinical RBE of C‐ion RT for low‐risk prostate cancer.

## 2 MATERIALS AND METHODS

### 2.1 Patient eligibility

The records of 262 low‐risk prostate cancer patients treated with C‐ion RT in QST Hospital in Chiba, Japan, between 2000 and 2018 were reviewed retrospectively. Eligible patients had a histological diagnosis of low‐risk adenocarcinoma of the prostate (clinical T1–2b, Gleason score ≤ 6, and initial prostate‐specific antigen (PSA) < 10 ng/ml), without evidence of lymph node or distant metastasis, and an Eastern Cooperative Oncology Group performance status of 0 or 1. The stage was determined based on the TNM classification 7th edition. Every patient received a digital rectal examination, magnetic resonance imaging (MRI), computed tomography (CT), and whole‐body bone scan to determine their clinical stage. All prostate biopsy samples were re‐evaluated by central pathologists. Patients who had ever received androgen deprivation therapy, pelvic RT, or treatment for prostate cancer or who experienced other malignancies during the last 5 years were excluded. This study was approved by the Ethical Review Committee of QST Hospital (approval code: 21–004; date of approval: May 14, 2021).

### 2.2 C‐ion RT

The method and technique for C‐ion RT for prostate cancer have been reported previously.^12^, ^16^ MRI was performed routinely soon after CT to produce fusion images. Three‐dimensional treatment planning was conducted using CT images of 3‐mm thickness fused to MR images. The clinical target volume included the whole prostate and proximal one‐third of the seminal vesicles. Planning target volume (PTV)1 was defined as the clinical target volume plus 10 mm margins in the anterior and lateral directions and 5 mm margins in other directions. Boost therapy was delivered to PTV2, delineated by the posterior edge in front of the anterior wall of the rectum and all other margins were the same as those of PTV1.

C‐ion RT was performed 4 days per week. Between 2000 and 2018, four different protocols were performed: 66 Gy (RBE) in 20 fractions (from 2000 to 2004), 63 Gy (RBE) in 20 fractions (from 2005 to 2007), 57.6 Gy (RBE) in 16 fractions (from 2007 to 2013), and 51.6 Gy (RBE) in 12 fractions (from 2013 to 2018), with fractional doses of 3.3 Gy (RBE), 3.15 Gy (RBE), 3.6 Gy (RBE), and 4.3 Gy (RBE), respectively.

### 2.3 Assessment and follow‐up

All patients were followed up by both the referring urologist and radiation oncologist at QST Hospital at 1 and 3 months after C‐ion RT, every 3 months during the first 3 years after C‐ion RT, and every 6 months thereafter. Acute and late toxicities were evaluated using the Common Terminology Criteria for Adverse Events version 4.0. The PSA nadir is defined as the lowest level of PSA after Carbon‐ion treatment. If the same PSA nadir level was seen in several time points, the first time point was recorded as the time of PSA nadir. Biochemical failure was defined, according to the Phoenix definition,^17^ as an increase in the PSA level greater than the PSA nadir plus 2 ng/ml. PSA density was defined as the PSA level divided by the prostate volume. Local failure was defined as radiological findings of disease recurrence at the primary site. Regional failure was defined as pelvic lymph node recurrence detected on imaging. Distant failure was defined as any metastasis to distant organs detected by radiographic examinations.

The primary endpoint was the biochemical or clinical failure‐free rate (BCFFR), defined as the percentage of patients who did not encounter any PSA failure or clinical evidence of failure (including local, regional, or distant) or receive any other salvage treatments. The follow‐up duration was calculated from the first day of C‐ion RT to the last follow‐up date. Cancer‐specific survival was based on deaths caused by prostate cancer. Overall survival was based on deaths from all causes during follow‐up duration.

### 2.4 PSA kinetics

The serum PSA levels at the start and end of C‐ion RT, and at every follow‐up, were collected. PSA bounce was defined as a ≥ 0.2 ng/ml increase in the PSA level above the last PSA nadir, followed by a return to the nadir, or below it, without additional treatment. PSA surge was defined as a rapid increase in the PSA level from initiation to completion of C‐ion RT. Strong (≥2.0 ng/ml increase) and weak (≥0.2 ng/ml increase) PSA surges were evaluated.

### 2.5 Statistical analysis

The BCFFR; local, regional, and distant failure‐free rates; and cancer‐specific survival were estimated by the Kaplan–Meier method. Univariate and multivariate Cox proportional‐hazards models were used to identify risk factors for biochemical or clinical failure. The time to PSA nadir was treated as a time‐dependent covariate. Differences in PSA level, PSA nadir, and PSA at 5 years of follow‐up among the four protocols were analyzed using the one‐way analysis of variance (ANOVA). Statistical analyses were performed using R (version R‐4.1.0; http://www.r‐project.org). A two‐sided *p* < 0.05 was considered statistically significant.

### 2.6 Tumor control probability (TCP) analysis

BCFFR is one form of the TCP. Previous eight references including long‐term follow‐up (at least 10 years) of low‐risk prostate cancer patients treated with definitive photon RT were collected for TCP analysis (Table S1). The total dose *D*, the number of fractions *n*, overall treatment time *T*, and 10‐year BCFFR were collected. The 10‐year BCFFR was plotted as a function of *D* and then fitted by the TCP model developed by Webb and Nahum (1993).
(1)

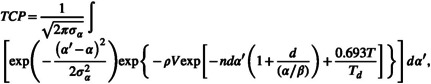

where *d* = *D*/*n* is the fraction size, *α* and *β* are the linear and quadratic coefficients of the linear‐quadratic model, *σ*_*α*_ is the standard deviation of the coefficient *α'* around *α*, representing patient‐to‐patient variation in radiosensitivity, *ρ* is the clonogenic cell density, *V* is the tumor volume, and *T*_d_ is the tumor doubling time.^18^ In the fitting, the values of *α* and *σ*_*α*_ in Equation (1) were changed in a step‐by‐step manner to best reproduce the clinical BCFFRs, while the values of *α*/*β*, *ρ*, *V*, and *T*_d_ were fixed to 1.5 Gy, 10^7^ cm^−3^, 1 cm^3^, and 365 days, respectively, as values typical for low‐risk prostate cancer treated with photon RT. With the adopted and determined values of *α*/*β*, *ρ*, *V*, *T*_d_, *α*, and *σ*_*α*_, the 10‐year BCFFR of low‐risk prostate cancer patients treated with photon RT can be estimated for any fractionation protocol.

The 10‐year BCFFRs for the C‐ion RT protocols of 66 Gy (RBE) in 20 fractions, 63 Gy (RBE) in 20 fractions, and 57.6 Gy (RBE) in 16 fractions obtained in our study were entered into the TCP analysis to derive dose–response parameters of low‐risk prostate cancer treated with C‐ion RT. Patients treated with 51.6 Gy (RBE) in 12 fractions was excluded from this analysis since the follow‐up duration was shorter than 10 years. The BCFFRs for the three protocols were plotted as a function of the absorbed dose. The absorbed dose was calculated as the RBE‐weighted clinical dose divided by the RBE of 2.41 at the center of a 60‐mm‐thick spread‐out Bragg peak C‐ion beam with a maximum energy of 350 MeV/μ, which is most frequently used for prostate cancer treatment. In the fitting, *ρ*, *V*, and *T*_d_ were unchanged from the fitting of photon data, while *α* and *α*/*β* of C‐ion RT were optimally determined to best reproduce the clinical BCFFRs of C‐ion RT. The value of *σ*_*α*_ was assumed to be 20% of *α*, following Kanai et al.^19^ Using the adopted and determined values of *ρ*, *V*, *T*_d_, *σ*_*α*_ *, α*, and *α*/*β*, the 10‐year BCFFR for low‐risk prostate cancer treated by C‐ion RT can be estimated for any fractionation protocol.

### 2.7 Clinical RBE calculation

Using Equation 1 with the values of *ρ*, *V*, *T*_d_, *σ*_*α*_ *, α*, and *α*/*β*, the dose resulting in a 10‐year BCFFR of 80% (*D*_80_) was determined for the 20, 16, and 12 fractionation protocols for photon and C‐ion RT. For each fractionation protocol, the clinical RBE of C‐ion RT for low‐risk prostate cancer was determined as the *D*_80_ of photon RT relative to the *D*_80_ of C‐ion RT.

For low‐risk prostate cancer, the ranges of the *ρ*, *V*, and *T*_d_ values were estimated to be 10^6^ to 10^8^ cm^−3^, 1–100 cm^3^, and 182–730 days, respectively. The ranges of *α*/*β* for photon RT and *σ*_*α*_ for C‐ion RT were estimated to be 1.5–2.5 Gy and 13–27% of *α*. The estimated range of the clinical RBE of C‐ion RT was derived by changing the parameter values of *ρ*, *V*, *T*_d_, *α*/*β*, and *σ*_*α*_ over the estimated ranges in the fitting of the BCFFRs for photon and C‐ion RTs.

## 3 RESULTS

### 3.1 Patient characteristics and treatment results

The pretreatment patient characteristics of the 262 eligible patients are listed in Table 1. The median age of the patients was 65 (range, 47–80) years. The median follow‐up was 8.4 (range, 0.9–18.4) years. Most patients had T1c (66.8%) and a Gleason score of 6 (95.4%).

**TABLE 1:** Patient characteristics

Characteristic	Number of patients (*N* = 262)
Age, median (range)	65 (47–80)
Follow‐up years, median (range)	8.36 (0.86–18.36)
T classification, *n* (%)	
T1a	1 (0.38%)
T1c	175 (66.80%)
T2a	83 (31.68%)
T2b	3 (1.15%)
Gleason score, *n* (%)	
4	1 (0.38%)
5	11 (4.20%)
6	250 (95.42%)
Initial PSA (ng/ml), median (range)	5.94 (2.46–9.96)
Prostate Volume (ml), median (range)	38.49 (19.63–113.72)
Treatment protocols, *n* (%)	
66Gy (RBE)/20fr	34 (12.98%)
63Gy (RBE)/20fr	29 (11.07%)
57.6Gy (RBE)/16fr	114 (43.51%)
51.6Gy (RBE)/12fr	85 (32.44%)

*Note*: Abbreviations: fr, fractions; Gy, Gray; PSA, Prostate‐specific Antigen; RBE, relative biological effectiveness.

**FIGURE 1:**
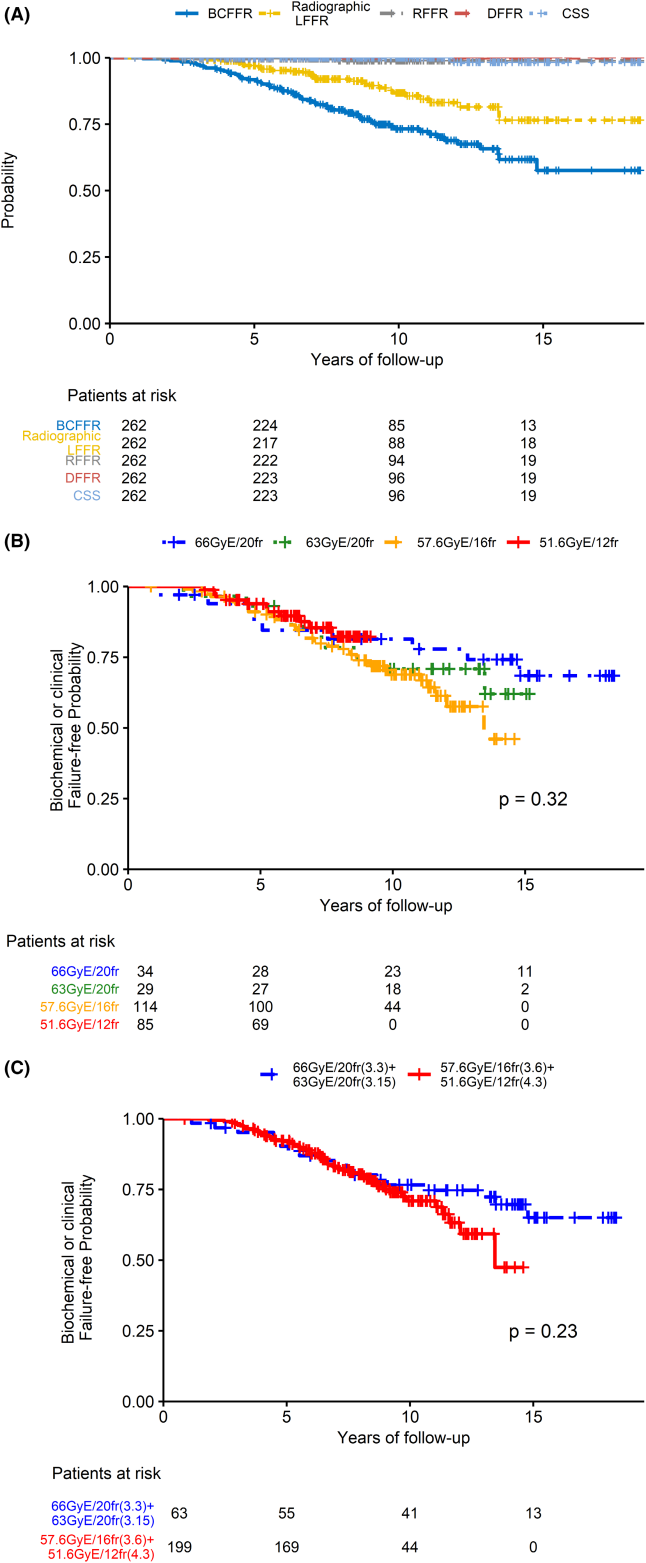
(A)The biochemical or clinical failure‐free rate (BCFFR), local‐failure rate, regional‐failure rate, distant‐failure rate, and the cancer‐specific survival of this study. (B)The BCFFR among 4 protocols (C)The BCFFR compared between lower and higher dose per fraction protocols.

The 10‐year BCFFR; local, regional, and distant failure‐free rates; cancer‐specific survival rate; and overall survival rate were 73.2%, 86.7%, 98.9%, 100%, 100%, and 89.3%, respectively (Figure 1A). The overall 5‐, 7‐, and 10‐year BCFFRs were 91.7% (95% CI, 88.4–95.22), 83.8% (95% CI, 79.3–88.66), and 73.2% (95% CI, 67.3–79.7), respectively. For patients who received C‐ion RT at 66 Gy (RBE) in 20 fractions, 63 Gy (RBE) in 20 fractions, or 57.6 Gy (RBE) in 16 fractions, the 10‐year BCFFRs were 81.4% (95% CI, 69.0–96.0), 70.9% (95% CI, 55.7–90.1), or 68.9% (95% CI, 60.3–78.7), respectively. There was no significant difference in the BCFFR among the four protocols estimated by the Kaplan–Meier method (*p =* 0.32) (Figure 1B). The follow‐up of the patients treated with 51.2 Gy (RBE) in 12 fractions was 7 years. There was a trend but no significant difference in the BCFFR of patients who received 66 or 63 Gy (RBE) in 20 fractions compared with 57.6 Gy (RBE) in 16 fractions or 51.2 Gy (RBE) in 12 fractions (*p =* 0.23) (Figure 1C).

There are 34 patients who received salvage treatment after biochemical recurrence as recorded. Among them, 23 patients (67.6%) received ADT, 6 patients (17.6%) received salvage radical prostatectomy, 3 patients (8.8%) received immune‐related therapy, 1 patient (1.5%) received re‐radiation of carbon‐ion therapy, and 1 patient (1.5%) received local cryotherapy. Of these 34 patients who received salvage therapy, only 1 patient developed regional progression, and 1 patient died due to prostate cancer.

### 3.2 PSA kinetics

**FIGURE 2:**
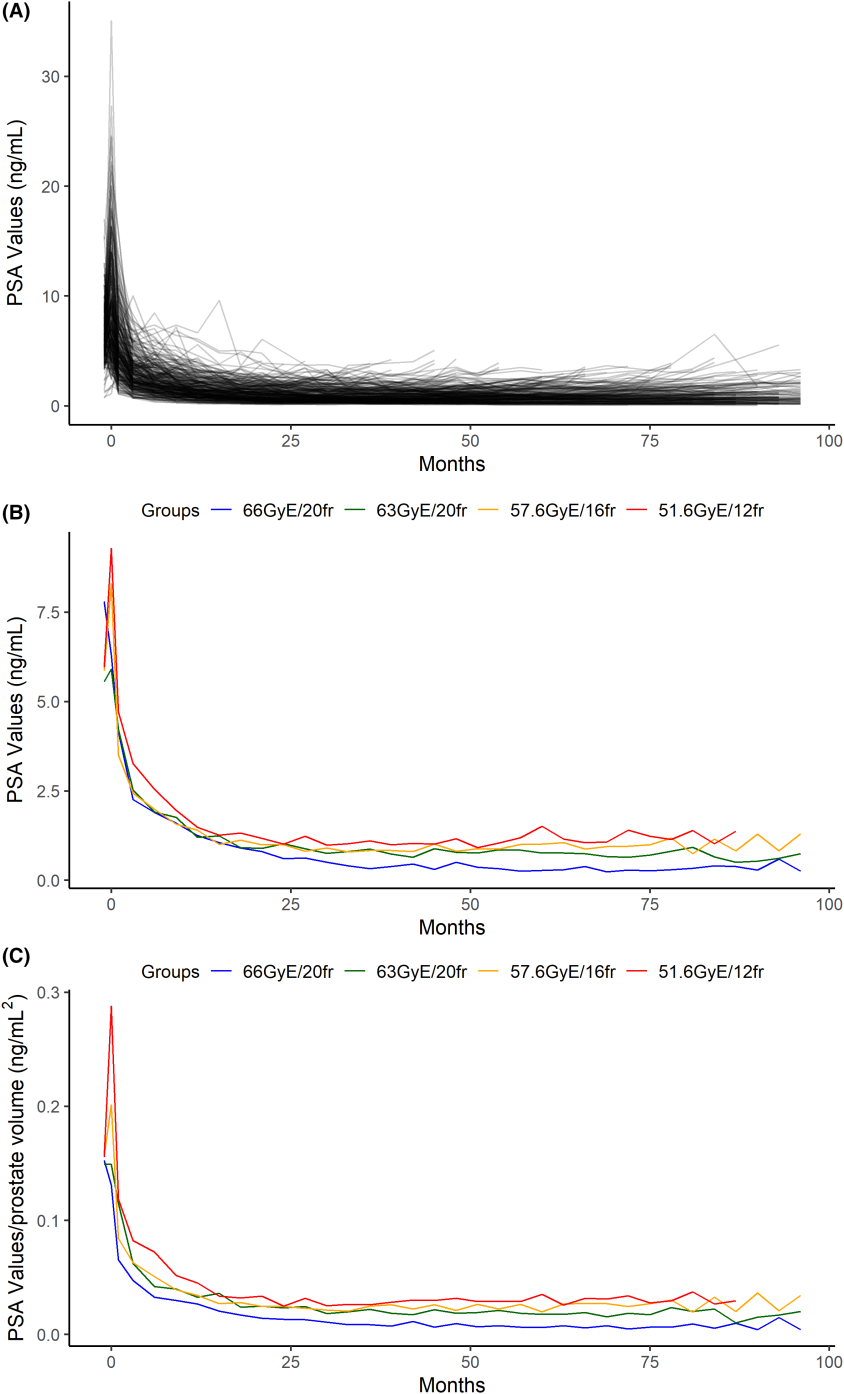
(A)The PSA kinetics after C‐ion RT for all patients (B)The median PSA level of four protocols during follow‐up (C)The median PSA‐Density of four protocols during follow‐up.

A total of 5127 PSA data were collected. The PSA kinetics after C‐ion RT of all patients are shown in Figure 2A. The median PSA level and density during follow‐up of the patients receiving each of the four protocols are presented in Figure 2B and C. There was a significant difference in the PSA level among the protocols (Table S2) around 2 years after C‐ion RT, and a significant difference in the PSA density among the protocols soon after C‐ion RT (Table S3). There were trends of a lower median PSA level and density after 66 Gy (RBE) in 20 fractions, whereas the median PSA level and density gradually increased after the other three protocols. The median PSA nadirs in the patients who received 66 Gy (RBE) in 20 fractions, 63 Gy (RBE) in 20 fractions, 57.6 Gy (RBE) in 16 fractions, and 51.6 Gy (RBE) in 12 fractions were 0.192 (range, 0.003–2.530), 0.495 (range, 0.060–1.760), 0.595 (range, 0.108–1.742), and 0.622 (range, 0.116–1.900) ng/ml, respectively. The respective times to nadir were 4.808 (range, 0.690–10.132), 3.451 (range, 0.918–12.274), 3.104 (range, 0.773–9.450), and 2.825 (range, 1.055–6.375) years (Table S4). Using the ANOVA, we found a statistically significant difference in PSA nadir and PSA level at 5 years among four protocols (*p* < 0.001). A subsequent comparison revealed that there is a significant difference in PSA nadir between patients who received protocols of 57.6 GyE/16fr and 66 GyE/20fr, and protocols of 51.6 GyE/16fr and 66 GyE/20fr, and a significant difference in PSA level at 5 years between patients who received protocols of 51.6 GyE/16fr and 66 GyE/20fr, and protocols of 51.6 GyE/16fr and 63 GyE/20fr. Figure S1 revealed the PSA nadir and PSA level at 5 years of follow‐up with the means and standard errors among four protocols.

A PSA bounce was observed in 48.9% (128/262) of patients after C‐ion RT. The BCFFR was significantly better in patients with a PSA bounce (*p* < 0.001). Patients who received 57.6 Gy (RBE) in 16 fractions and 51.6 Gy (RBE) in 12 fractions had an obvious PSA surge compared with the other two protocols. A weak PSA surge (≥0.2 ng/ml) was seen in 53.1% (139/262) of patients and a strong surge (≥2.0 ng/ml) in 36.6% (96/262) of patients. There was no association between the BCFFR and PSA surge, regardless of the cutoff PSA level used (0.2 vs. 2.0 ng/ml) (Figure S2).

### 3.3 Tumor control probability (TCP) analysis

**FIGURE 3:**
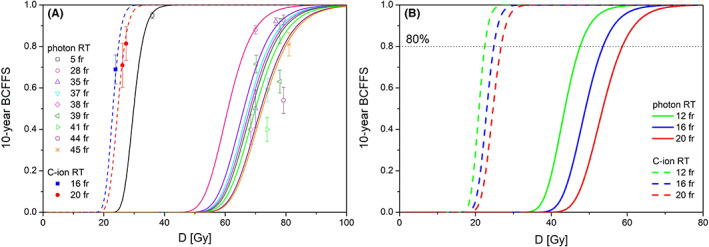
(A) The 10‐year biochemical or clinical failure‐free rate (BCFFR) of low‐risk prostate cancer treated by photon radiotherapy (RT) (open squares), and those treated by carbon‐ion (C‐ion) RT (closed circles). The solid curves are predicted Tumor control probability (TCP) curves of photon RT with the optimum values of α = 0.112 ± 0.001 Gy^−1^ and σ_α_ = 0.017 ± 0.001 Gy^−1^, while the dashed curves are the predicted TCP curves of C‐ion RT with the optimum values of α = 0.394 ± 0.018 Gy^−1^ and α/β = 1.71 ± 0.11 Gy. (B) The predicted TCP curves of low‐risk prostate cancer treated by photon RT (solid curves) and C‐ion RT (dashed curves). The dotted line shows the 10‐year BCFFR of 80%. The clinical RBE of C‐ion RT is given by the D80 of photon RT divided by the D80 of C‐ion RT for the respective fractions.

The optimal *α* and *σ*_*α*_ values for low‐risk prostate cancer treated with photon RT were 0.112 ± 0.001 Gy^−1^ and 0.017 ± 0.001 Gy^−1^, respectively. The TCP curves calculated using the parameter values reasonably reproduced the reported 10‐year BCFFRs of patients treated with various fractionation protocols of photon RT, as shown in Figure 3A. The optimal *α* and *α*/*β* values for C‐ion RT were 0.394 ± 0.018 Gy^−1^ and 1.71 ± 0.11 Gy, respectively, which accurately reproduced the 10‐year BCFFRs of patients treated with the three fractionation protocols of C‐ion RT (Figure 3A).

### 3.4 Clinical RBE

The estimated *D*_80_ values of photon RT were 58.7 (range, 58.6–60.0) Gy for 20 fractions, 53.6 (range, 53.5–55.3) Gy for 16 fractions, and 47.6 (range, 47.4–49.6) Gy for 12 fractions. The estimated *D*_80_ values of C‐ion RT were 26.8 (range, 26.7–27.0) Gy for 20 fractions, 24.8 (range, 24.5–25.0) Gy for 16 fractions, and 22.5 (range, 21.8–22.8) Gy for 12 fractions, which corresponded to the fixed RBE‐weighted clinical doses of 64.5 (range, 64.3–65.0) Gy (RBE), 59.9 (range, 59.0–60.3) Gy (RBE), and 54.1 (range, 52.6–54.8) Gy (RBE), respectively. The clinical RBE of C‐ion RT for 20, 16, and 12 fractions were 2.19 (2.18–2.24), 2.16 (2.14–2.23), and 2.12 (2.09–2.21), respectively (Figure 3B). The clinical RBE of C‐ion RT decreased when the dose per fraction increased.

### 3.5 Risk factor assessment

Higher PSA nadir (*p* < 0.001), shorter time to PSA nadir (*P* < 0.001), smaller prostate volume (*p* = 0.007), and lack of a PSA bounce (*p* < 0.001) were identified as risk factors for biochemical or clinical failure in univariate Cox regression analyses (Table 2). In the multivariate Cox regression analysis (Table 2), a higher PSA nadir (*p* < 0.001) remained a significant risk factor for biochemical or clinical failure, and a shorter time to PSA nadir was a significant risk factor during 2–10 years of follow‐up. The multivariate Cox regression results for the time to PSA nadir every 2 years during follow‐up are provided (Table S5). There was no significant difference in the BCFFR according to protocol, PSA surge, initial PSA level, or age.

**TABLE 2:** The hazard ratio and *p* value of each factor in univariate and multi‐variate Cox model for biochemical or clinical failure‐free rate in low‐risk prostate cancer patients after Carbon‐ion Radiotherapy

Factor	Univariate analysis		Multi‐variate analysis	
	Hazard ratio (95% CI)	*p* value	Hazard ratio (95% CI)	*p* value
**Protocols**				
66 Gy (RBE)/20fr	Reference		—	—
63 Gy (RBE)/20fr	1.381 (0.527–3.620)	0.511		
57.6 Gy (RBE)/16fr	1.852 (0.813–4.216)	0.142		
51.6 Gy (RBE)/12fr	1.203 (0.440–3.288)	0.719		
PSA nadir value (ng/ml)	4.748 (2.672–8.435)	<0.001	1.85 (1.065–3.228)	0.029
Time to PSA nadir (years)	0.451 (0.352–0.577)	<0.001	—^a^	<0.05
Prostate volume (ml)	0.973 (0.956–0.991)	0.002	0.978 (0.959–0.998)	0.031
PSA bounce				
With	0.422 (0.255–0.699)	<0.001	0.752 (0.427–1.330)	0.323
Without	Reference			
PSA surge				
With	0.844 (0.493–1.443)	0.534	—	—
Without	Reference			
Initial PSA level	1.006 (0.880–1.150)	0.933	—	—
Age	0.993 (0.962–1.025)	0.658	—	—

*Note*: Abbreviations: CI, confidence interval; fr, fractions; PSA, Prostate‐specific Antigen; RBE, relative biological effectiveness.

^a^ *p* < 0.05 was noted during 2–10 years of follow‐up. Time to PSA nadir is a time covariate, and the Hazard ratio and *p* value of time to PSA nadir in multi‐variate Cox model was provided in Table S5.

### 3.6 Toxicity

There was no grade 3 or 4 gastrointestinal (GI), genitourinary (GU), or skin toxicities among all patients (Table 3). The rate of early and late grade 2 GU toxicities in the whole cohort were 8.8% and 7.6%. The rates of early and late grade 2 GI toxicities were 0.4% and 1.5%, respectively. There was no grade 2 early or late skin toxicities among all patients.

**TABLE 3:** Toxicity of different protocols

No.	Early or late	Grade	66 Gy (RBE)/20fr (3.3 Gy (RBE)/fr) *n* = 34	63 Gy (RBE)/20fr (3.15 (RBE)/fr) *n* = 29	57.6 Gy (RBE)/16fr (3.6 Gy (RBE)/fr) *n* = 114	51.6 Gy (RBE)/12fr (4.3 Gy (RBE)/fr) *n* = 85	Total *n* = 262
GU toxicity^a^, *n* (%)	Acute	1	5 (17.14%)	4 (13.79%)	66 (57.89%)	31 (36.47%)	106 (40.46%)
		2	0 (0%)	1 (3.45%)	10 (8.77%)	12 (14.12%)	23 (8.78%)
	Late	1	**24 (68.57%)**	**22 (75.86%)**	**72 (63.16%)**	**20 (23.53%)**	**138** (52.67%)
		2	**8 (25.71%)**	**3 (10.32%)**	**5 (4.39%)**	**4 (4.71%)**	**20 (7.63%)**
GI Toxicity^a^, *n* (%)	Acute	1	0 (0%)	0 (0%)	2 (1.75%)	6 (7.06%)	8 (3.05%)
		2	0 (0%)	0 (0%)	1 (0.88%)	0 (0%)	1 (0.38%)
	Late	1	7 (20.00%)	2 (6.90%)	6 (5.26%)	9 (10.59%)	24 (9.16%)
		2	3 (8.57%)	0 (0%)	0 (0%)	1 (1.18%)	4 (1.53%)
Skin Toxici*t*y^a^, *n* (%)	Acute	1	0 (0%)	2 (6.90%)	5 (4.39%)	4 (4.71%)	11 (4.20%)
		2	0 (0%)	0 (0%)	0 (0%)	0 (0%)	0 (0%)
	Late	1	1 (2.86%)	0 (0%)	1 (0.88%)	0 (0%)	2 (0.76%)
		2	0 (0%)	0 (0%)	0 (0%)	0 (0%)	0 (0%)

*Note*: Abbreviations: fr, fractions; GI, Gastrointestinal; GU, Genitourinary.

^a^ There is no grade 3 or 4 toxicity in this study.

## 4 DISCUSSION

This is the first study to use clinical data to evaluate the clinical RBE of C‐ion RT. Our results showed that the RBE of C‐ion beams decreased when the dose per fraction increased. Patients who received 66 Gy (RBE) in 20 fractions had a better 10‐year BCFFR, lower PSA nadir, and longer time to reach PSA nadir, compared with other protocols. A higher PSA nadir and shorter time to reach PSA nadir were risk factors for biochemical or clinical failure.

In proton RT, a generic RBE value of 1.1 has been used for all different tumors and fraction sizes.^20^ For C‐ion RT in Japan, a fixed clinical RBE system, in which the RBE is dependent on the depth of target but independent of the tumor type or fraction size, has been used for clinical treatment.^21^ However, several preclinical studies showed that the RBE usually decreases with increasing dose per fraction, especially in cancer types with low α/β values.^11^ Paganetti et al. reported a 5–10% higher RBE of proton RT for 2 Gy (RBE) than for 6 Gy (RBE).^10^ Khachonkham et al. reported a difference (13%) between 2 Gy (RBE) and 4 Gy (RBE) in prostate cancer cell lines,^11^ and a similar phenomenon was also found in preclinical C‐ion RT studies.^22^, ^23^

The randomized controlled trials PROFIT, CHHiP, and RTOG0415 showed that hypofractionated RT was not inferior to conventional RT in prostate cancer.^24^, ^25^, ^26^ Therefore, a trend of using hypofractionation has developed. Based on the linear‐quadratic model and assuming that the α/β of prostate cancer cells is 1.5, 66 Gy (RBE) in 20 fractions, 63 Gy (RBE) in 20 fractions, 57.6 Gy (RBE) in 16 fractions, and 51.6 Gy (RBE) in 12 fractions had similar equivalent dose at fractionation of 2 Gy (EQD2) values of 90.5, 83.7, 83.9, and 85.5 Gy (RBE), respectively. However, our TCP analyses revealed that the 10‐year BCFFRs estimated for the four protocols were 86% (range, 81%–90%), 73% (range, 63%–82%), 66% (range, 58%–75%), and 61% (range, 56%–68%), respectively, and fixed RBE‐weighted clinical doses of 64.5, 59.9, and 54.1 Gy (RBE) were required to achieve a 10‐year BCFFR of 80% in the 20, 16, and 12 fractionation protocols, respectively. In the TCP analyses, the clinical RBE of C‐ion RT for 20, 16, and 12 fractions in low‐risk prostate cancer were 2.19 (2.18–2.24), 2.16 (2.14–2.23), and 2.12 (2.09–2.21), respectively. A larger dose per fraction resulted in a lower clinical RBE.

PSA is a tumor marker widely used for monitoring prostate cancer.^27^, ^28^ Since the PSA level is related to the prostate volume, the PSA density, has been used to define the clinical effectiveness of RT.^29^, ^30^ Our results showed a difference among the four protocols in the PSA level and PSA density after C‐ion RT. The lower dose per fraction protocols resulted in a lower PSA level and density compared with the higher dose per fraction protocols. These results suggest that the prescribed doses for the 16 and 12‐fraction protocols should be escalated, despite similar EQD2 values to those of the 20‐fraction protocols.

In our study, a higher PSA nadir was a risk factor for biochemical or clinical failure, and a shorter time to PSA nadir was a risk factor at 2–10 years of follow‐up. Less than 2 years may be too short for the development of low‐risk prostate cancer, and the number of patients was limited after 10 years of follow‐up. Geara et al. found that a PSA nadir <0.06 ng/ml results in better biochemical survival.^31^ Pile et al. showed a PSA nadir ≥0.2 ng/ml and a time to nadir <12 months were associated with an increased mortality.^32^ These findings support our results.

Our study showed a significantly better BCFFR in the patients with a PSA bounce, which may be related to a higher immune response, contributing to better tumor control.^33^, ^34^ A PSA surge after C‐ion RT was observed, and PSA surge was not related to the BCFFR. A similar phenomenon was reported by Darwis et al.^34^ The smaller prostate size was a borderline risk factor in this study. Freedland et al. showed that a prostate weight < 20 g conferred an 8.43 times higher risk of biochemical progression compared with a prostate weight ≥ 100 g,^35^ possibly because a small prostate has a low androgen concentration, allowing only more aggressive cancers to grow.^35^ Although the mechanism is still unclear, these results are in accordance with our findings.

The rate of grade ≥ 2 late GU toxicities was approximately 13–40% after photon RT,^36^, ^37^ and was 7.6% in our study. The rate of grade ≥ 2 late GI toxicities was approximately 12–26% after photon RT,^38^ and was only 1.5% in our study, consistent with previous studies.^5^, ^15^ The low rate of side effects after C‐ion RT facilitates clinical trials evaluating further hypofractionation protocols. Based on TCP analysis using the parameter values determined in this study, the estimated *D*_80_ value for the 4 fraction treatments was 14.6 (range, 13.7–15.2) Gy, which corresponds to the fixed RBE‐weighted clinical dose of 35.3 (range, 32.9–36.6) Gy (RBE). Following the analyses, at QST Hospital, dose‐escalation clinical trials of C‐ion RT using 36, 38, and 40 Gy (RBE) in 4 fractions have commenced. In the clinical trials, in order to reduce the risk of urethral stenosis, the dose to the urethra has been reduced to 90% of the protocol dose, i.e., 32.6–36 Gy (RBE).

There are some limitations to this study. First, this was a retrospective study conducted in a single institute. Second, the C‐ion RT technique evolved from passive to scanning irradiation method during the study period, but no significant difference in dose quality was found as reported in the previous studies.^39^ Third, a longer follow‐up is needed considering the slow‐growing nature of prostate cancer. Forth, only the statistical error was considered for the 10‐year BCFFSs used for the analyses, by neglecting other errors pertaining to setup and irradiation in each treatment. Fifth, only three 10‐year BCFFS data were used to estimate the TCP parameters of C‐ion RT, and the data should be reanalyzed including the 10‐year BCFFS for 12 fractions in the future.

In conclusion, utilizing clinical data from individuals with low‐risk prostate cancer, we defined clinical RBE in C‐ion RT. The clinical RBE of C‐ion RT reduced with increasing dose per fraction. Future cross‐institutional research of the clinical outcomes after carbon‐ion therapy of diverse dose‐fractionation protocols is required, and new clinical trials of C‐ion RT have begun for further investigation in light of our findings.

### AUTHOR CONTRIBUTION

Yu‐Mei Kang: conceptualization, data curation, formal analysis, investigation, methodology, software, validation, visualization, writing ‐ original draft, and writing ‐ review and editing. Hitoshi Ishikawa: conceptualization, data curation, formal analysis, funding acquisition, investigation, methodology, project administration, resources, supervision, validation, writing ‐ original draft, and writing ‐ review and editing. Taku Inaniwa: conceptualization, formal analysis, investigation, methodology, software, validation, visualization, writing ‐ original draft, and writing ‐ review and editing. Yuma Iwai: data curation, investigation, methodology, project administration, resources, and writing ‐ review and editing. Naruhiro Matsufuji: methodology, resources, supervision, and writing ‐ review and editing. Goro Kasuya: data curation, methodology, resources, and writing ‐ review and editing. Noriyuki Okonogi: data curation, investigation, methodology, project administration, resources, and writing ‐ review and editing. Yu‐Ming Liu: resources, supervision, and writing ‐ review and editing; Yee Chao: resources, supervision, and writing ‐ review and editing; Masaru Wakatsuki: data curation, resources, supervision, and writing ‐ review and editing; Hirohiko Tsujii: data curation, resources, supervision, and writing ‐ review and editing; Hiroshi Tsuji: data curation, resources, supervision, and writing ‐ review and editing.

## FUNDING INFORMATION

This work was supported by Grants‐in‐Aid for Scientific Research (C) (21K07715) from the Ministry of Education, Culture, Sports, Science and Technology of Japan. Dr. Yu‐Mei, Kang was supported by Yin Shu‐Tien Foundation Taipei Veterans General Hospital‐National Yang‐Ming University Excellent Physician Scientists Cultivation Program 109‐V‐A‐008 to receive training in QST Hospital.

## CONFLICT OF INTEREST

None.

## ETHICS STATEMENT

This study was approved by the Ethical Review Committee of QST Hospital (approval code: 21–004; date of approval: May 14, 2021).

## Supporting information


Appendix S1

## Data Availability

The data that support the findings of this study are available from the corresponding author upon reasonable request.
