# Supplementary material for: The clinical relative biological effectiveness and prostate‐specific antigen kinetics of carbon‐ion radiotherapy in low‐risk prostate cancer
Source: Cancer Med. 2022 Jul 19;12(2):1540–51. doi: 10.1002/cam4.5045 (PMC9883571; doi:10.1002/cam4.5045)
Supplement: Supplementary file 1 — Appendix S1 [file CAM4-12-1540-s001.pdf]

Supplementary Material

Supplementary Fig. S1

(a) The PSA nadir with the means and standard errors among four protocols.

(b) The PSA level at 5 years of follow-up with the means and standard errors among four protocols.

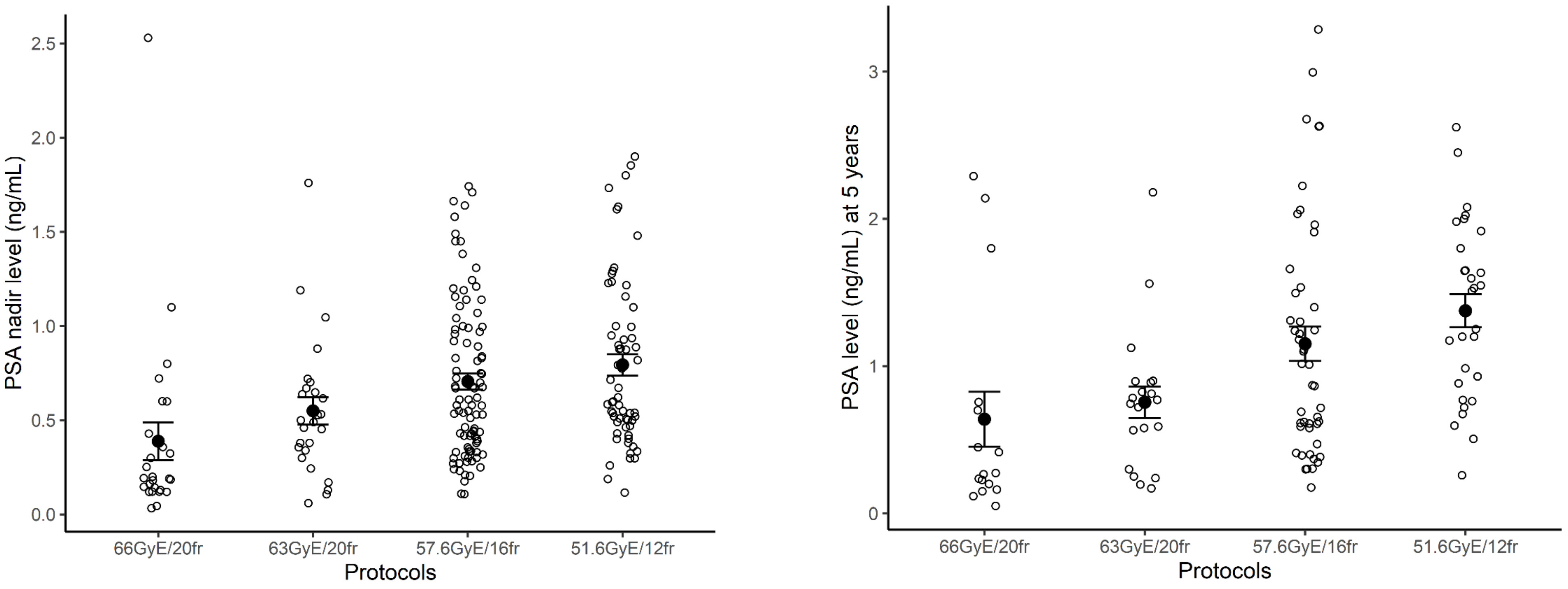

† **Abbreviations:** PSA: Prostate-specific Antigen

Supplementary Fig. S2

(a) The biochemical or clinical failure-free rate compared patients with a PSA bounce and without a PSA bounce (PSA bounce cutting point: 0.2 ng/mL).

(b) The biochemical or clinical failure-free rate compared patients with a PSA surge and without a PSA surge (PSA surge cutting point: 0.2 ng/mL).

(c) The biochemical or clinical failure-free rate compared patients with a PSA surge and without a PSA surge (PSA surge cutting point: 2.0 ng/mL).

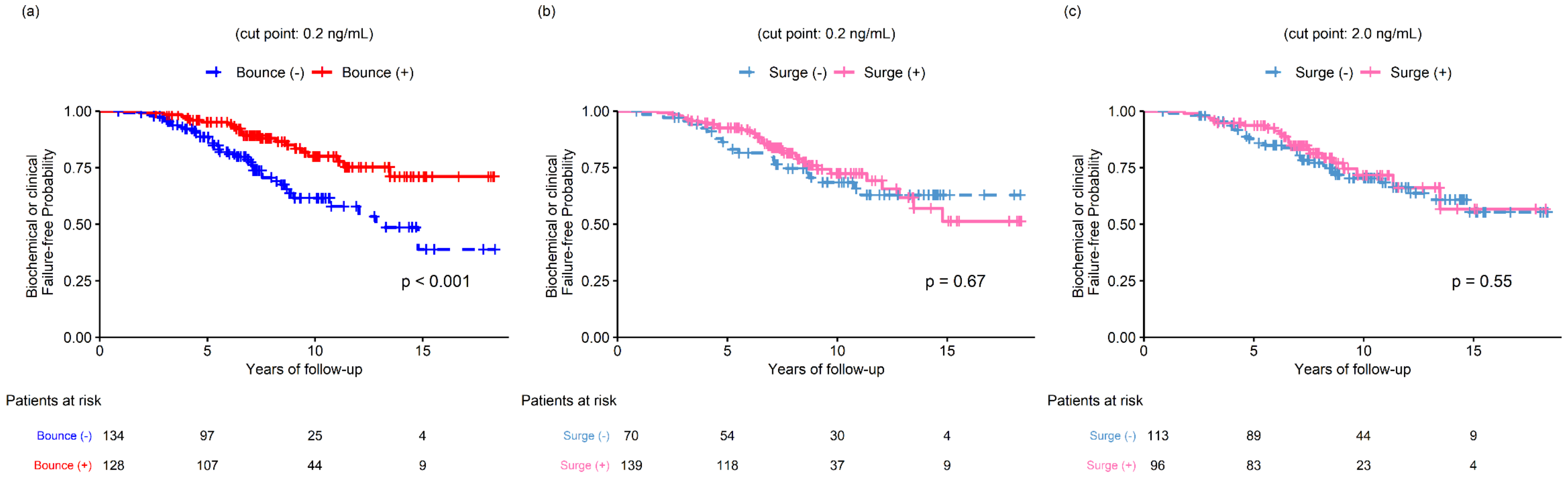

† **Abbreviations:** PSA: Prostate-specific Antigen

Supplementary Table S1. Reference used in Tumor control probability model analysis

**Study type**	**Total patient numbers**	**Low-risk patient numbers**	**Median follow-up (years)**	**Protocols (Total dose/ fractions)**	**Treatment period (weeks)**	**7-year biochemical or clinical failure-free rate (low-risk only)**	**7-year biochemical or clinical failure-free rate (Whole cohort)**	**8-year biochemical or clinical failure-free rate (low-risk only)**	**8-year biochemical or clinical failure-free rate (Whole cohort)**	**10-year biochemical or clinical failure-free rate (low-risk only)**	**10-year biochemical or clinical failure-free rate (Whole cohort)**	**ADT (low-risk)**	**Risk classification**	**Failure definition**	**Reference**
Randomized	206	29<br>28	8.5	75.6Gy/42fr<br>72Gy/30fr	8.4<br>6	-	**85%**<br>**90.5%**	-	**84.6%**<br>**89.3%**	-	**76.3%**<br>**89.3%**	24%<br>(+)	NCCN	Phoenix	1
Randomized	862	79<br>81	10.0	74Gy/37fr<br>64Gy/32fr	7.4<br>6.4	-	**65%**<br>**50%**	-	**62.5%**<br>**49.5%**	-	**55%**<br>**43%**	100%<br>(+)	NCCN	Phoenix	2
Randomized	393	111<br>116	8.9	70.2Gy/39fr<br>79.2Gy/44fr	7.8<br>8.8	**73%**<br>**93.8%**	**78.5%**<br>**91.8%**	**73%**<br>**92.8%**	**75%**<br>**91.8%**	**71.5%**<br>**92.8%**	**68%**<br>**82.6%**	(-)	NCCN	Phoenix	3
Randomized	3216	157<br>164<br>163	5.2	74Gy/37fr<br>60Gy/20fr<br>57Gy/19fr	7.4<br>4<br>3.8	**95%**<br>**92%**<br>**86%**	**84%**<br>**84%**<br>**78%**	-	-	-	-	optional	NCCN	Phoenix	4
Randomized	1092	542<br>550	5.8	73.8Gy/41fr<br>70Gy/28fr	8.2<br>5.6	**88%**<br>**92%**	**88%**<br>**92%**	-	-	-	-	(-)	-	Phoenix	5
Randomized	126	25	13.7	74Gy/37fr<br>64Gy/32fr	7.4<br>6.4	-	**62%**<br>**54%**	-	**58%**<br>**45%**	-	**49%**<br>**45%**	100%<br>(+)	-	two rises in PSA> 2ng/ml	6
Retrospective	854	266	11.3	70Gy/28fr	5.6	**94%**	**77%**	**92%**	**75%**	**88%**	**72%**	(-)	NCCN	Phoenix	7
Retrospective	230	230	9	35-36Gy/5fr	1	**97.2%**	**97.2%**	**95%**	**95%**	**95%**	**93.7%**	(-)	NCCN	Phoenix	8
Retrospective	170	49	8.25	81Gy/45ffr	9	**91%**	-	**88%**	-	**81%**	-	(-)	NCCN	Phoenix	9
Retrospective	301	31<br>30	8.7	70Gy/35fr<br>78Gy/39fr	8.7	**77.6%**<br>**93.8%**	**70%**<br>**80%**	**62.5%**<br>**87.5%**	**58.8%**<br>**77.5%**	**50%**<br>**87.5%**	**50%72.5%**	(-)	NCCN	Phoenix	10
Retrospective	596	226	5.2	76.7Gy/35fr	7	**97%**	**90%**	**92%**	**87%**	**92%**	**87%**	21.2%	D'Amico	Phoenix	11

† **Abbreviations:** Gy: Gray; fr: fractions; ADT: androgen deprivation therapy

Reference:

1. Hoffman KE, Voong KR, Levy LB, et al. Randomized Trial of Hypofractionated, Dose-Escalated, Intensity-Modulated Radiation Therapy (IMRT) Versus Conventionally Fractionated IMRT for Localized Prostate Cancer. *J Clin Oncol*. Oct 10 2018;36(29):2943-2949. doi:10.1200/jco.2018.77.9868
2. Dearnaley DP, Jovic G, Syndikus I, et al. Escalated-dose versus control-dose conformal radiotherapy for prostate cancer: long-term results from the MRC RT01 randomised controlled trial. *Lancet Oncol*. Apr 2014;15(4):464-73. doi:10.1016/s1470-2045(14)70040-3
3. Zietman AL, Bae K, Slater JD, et al. Randomized trial comparing conventional-dose with high-dose conformal radiation therapy in early-stage adenocarcinoma of the prostate: long-term results from proton radiation oncology group/american college of radiology 95-09. *J Clin Oncol*. Mar 1 2010;28(7):1106-11. doi:10.1200/jco.2009.25.8475
4. Dearnaley D, Syndikus I, Mossop H, et al. Conventional versus hypofractionated high-dose intensity-modulated radiotherapy for prostate cancer: 5-year outcomes of the randomised, non-inferiority, phase 3 CHHiP trial. *Lancet Oncol*. Aug 2016;17(8):1047-1060. doi:10.1016/s1470-2045(16)30102-4
5. Lee WR, Dignam JJ, Amin MB, et al. Randomized Phase III Noninferiority Study Comparing Two Radiotherapy Fractionation Schedules in Patients With Low-Risk Prostate Cancer. *J Clin Oncol*. Jul 10 2016;34(20):2325-32. doi:10.1200/jco.2016.67.0448
6. Creak A, Hall E, Horwich A, et al. Randomised pilot study of dose escalation using conformal radiotherapy in prostate cancer: long-term follow-up. *Br J Cancer*. Aug 6 2013;109(3):651-7. doi:10.1038/bjc.2013.394
7. Abu-Gheida I, Reddy CA, Kotecha R, et al. Ten-Year Outcomes of Moderately Hypofractionated (70 Gy in 28 fractions) Intensity Modulated Radiation Therapy for Localized Prostate Cancer. *Int J Radiat Oncol Biol Phys*. Jun 1 2019;104(2):325-333. doi:10.1016/j.ijrobp.2019.01.091
8. Katz A. Stereotactic Body Radiotherapy for Low-Risk Prostate Cancer: A Ten-Year Analysis. *Cureus*. Sep 9 2017;9(9):e1668. doi:10.7759/cureus.1668
9. Alicikus ZA, Yamada Y, Zhang Z, et al. Ten-year outcomes of high-dose, intensity-modulated radiotherapy for localized prostate cancer. *Cancer*. 2011;117(7):1429-1437. doi:https://doi.org/10.1002/cncr.25467
10. Kuban DA, Tucker SL, Dong L, et al. Long-term results of the M. D. Anderson randomized dose-escalation trial for prostate cancer. *Int J Radiat Oncol Biol Phys*. Jan 1 2008;70(1):67-74. doi:10.1016/j.ijrobp.2007.06.054
11. Teh BS, Lewis GD, Mai W, Pino R, Ishiyama H, Butler EB. Long-term outcome of a moderately hypofractionated, intensity-modulated radiotherapy approach using an endorectal balloon for patients with localized prostate cancer. *Cancer Commun (Lond)*. Apr 17 2018;38(1):11. doi:10.1186/s40880-018-0281-4

Supplementary Table S2. The difference of PSA level after Carbon-ion Radiotherapy during different follow-up time points

Follow-up months	p value of one-way ANOVA
C-ion RT Start	<0.001
C-ion RT End	0.001
1	0.101
3	0.092
6	0.089
9	0.518
12	0.308
15	0.297
18	0.316
21	0.199
24	0.044
27	0.011
30	0.114
33	0.006
36	0.008
39	0.013
42	0.068
45	0.340
48	0.184
51	0.040
54	0.100
57	0.006
60	0.001
63	0.011
66	0.086
69	0.002
72	0.006
75	0.019
78	0.052
81	0.033
84	0.038
87	0.326
90	0.026
93	0.559
96	0.001

† **Abbreviations:** C-ion RT: Carbon-ion Radiotherapy; ANOVA: Analysis of variance

Supplementary Table S3. The difference of PSA density (PSA level divided by prostate volume) during different follow-up time points

Follow-up months	p value of one-way ANOVA
C-ion RT Start	0.413
C-ion RT End	<0.001
1	0.053
3	0.001
6	0.001
9	0.098
12	0.049
15	0.061
18	0.059
21	0.026
24	0.009
27	0.001
30	0.028
33	0.001
36	0.002
39	0.004
42	0.005
45	0.076
48	0.038
51	0.004
54	0.008
57	<0.001
60	<0.001
63	0.002
66	0.005
69	0.001
72	0.002
75	0.011
78	0.025
81	0.012
84	0.024
87	0.089
90	0.002
93	0.675
96	0.004

† **Abbreviations:** C-ion RT: Carbon-ion Radiotherapy; ANOVA: Analysis of variance

Supplementary Table S4. Median PSA nadir and time to PSA nadir of different protocols

Protocols	Median PSA nadir (ng/mL) (range)	Time to PSA nadir (years) (range)
66 Gy (RBE) / 20fr	0.192 (0.003-2.530)	4.808 (0.690-10.132)
63 Gy (RBE) / 20fr	0.495 (0.060-1.760)	3.451 (0.918-12.274)
57.6 Gy (RBE) / 16fr	0.595 (0.108-1.742)	3.104 (0.773-9.450)
51.6 Gy (RBE) / 12fr	0.622 (0.116-1.900)	2.825 (1.055-6.375)

† **Abbreviations:** Gy: Gray; RBE: relative biological effectiveness; fr: fractions; PSA: Prostate-specific Antigen

Supplementary Table S5. The hazard ratio and *P* value of each factor in univariate and multi-variate Cox model for biochemical or clinical failure-free rate in low-risk prostate cancer patients after Carbon-ion Radiotherapy

**Factors**		**Multi-variate analysis**	
		**Hazard ratio (95% CI)**	***P* value**
**Nadir (ng/mL)**		1.854 (1.065-3.228)	0.029
**Reach nadir time (years)**	**Follow-up year**		
	**0-2**	<0.01 (<0.01-9.590)	0.112
	**2-4**	0.116 (0.035-0.390)	<0.001
	**4-6**	0.352 (0.202-0.653)	<0.001
	**6-8**	0.550 (0.371-0.614)	<0.01
	**8-10**	0.561 (0.367-0.825)	<0.01
	**10-12**	0.708 (0.637-0.856)	0.1888
	**>12**	0.808 (0.424-1.184)	0.315
**Prostate volume (mL)**		0.978 (0.959-0.998)	0.031
**PSA bounce**		0.752 (0.427-1.323)	0.323

† **Abbreviations:** PSA: Prostate-specific Antigen; CI: confidence interval
